# Molecular Mechanisms of Action of Selected Substances Involved in the Reduction of Benzo[a]pyrene-Induced Oxidative Stress

**DOI:** 10.3390/molecules27041379

**Published:** 2022-02-18

**Authors:** Bożena Bukowska, Piotr Duchnowicz

**Affiliations:** Department of Biophysics of Environmental Pollution, Faculty of Biology and Environmental Protection, University of Lodz, Pomorska Str. 141/143, 90-236 Lodz, Poland; piotr.duchnowicz@biol.uni.lodz.pl

**Keywords:** benzo(a)pyrene, oxidative stress, antioxidative properties, polyphenols, vitamin E, curcumin, green and white tea, taurine, atorvastatin, diallyl sulfide

## Abstract

Benzo[a]pyrene (BaP) is a polycyclic aromatic hydrocarbon (PAH) primarily formed by burning of fossil fuels, wood and other organic materials. BaP as group I carcinogen shows mutagenic and carcinogenic effects. One of the important mechanisms of action of (BaP) is its free radical activity, the effect of which is the induction of oxidative stress in cells. BaP induces oxidative stress through the production of reactive oxygen species (ROS), disturbances of the activity of antioxidant enzymes, and the reduction of the level of non-enzymatic antioxidants as well as of cytokine production. Chemical compounds, such as vitamin E, curcumin, quercetin, catechin, cyanidin, kuromanin, berberine, resveratrol, baicalein, myricetin, catechin hydrate, hesperetin, rhaponticin, as well as taurine, atorvastatin, diallyl sulfide, and those contained in green and white tea, lower the oxidative stress induced by BaP. They regulate the expression of genes involved in oxidative stress and inflammation, and therefore can reduce the level of ROS. These substances remove ROS and reduce the level of lipid and protein peroxidation, reduce formation of adducts with DNA, increase the level of enzymatic and non-enzymatic antioxidants and reduce the level of pro-inflammatory cytokines. BaP can undergo chemical modification in the living cells, which results in more reactive metabolites formation. Some of protective substances have the ability to reduce BaP metabolism, and in particular reduce the induction of cytochrome (CYP P450), which reduces the formation of oxidative metabolites, and therefore decreases ROS production. The aim of this review is to discuss the oxidative properties of BaP, and describe protective activities of selected chemicals against BaP activity based on of the latest publications.

## 1. Introduction

Benzo[a]pyrene (BaP) is a polycyclic aromatic hydrocarbon (PAH) found in cigarette smoke, grilled meats, and byproducts of industrial incineration. BaP bioaccumulates in organisms and is formed De Novo by certain food processing methods, such as smoking and cooking in high temperatures [1,2]. Major sources for human exposure to BaP are contaminated food [3], water [4], and air [5]. BaP metabolite: 7β,8α-dihydroxy-9α,10α-epoxy-7,8,9,10-tetrahydrobenzo[a]pyrene (BPDE-I), forms adducts with DNA (anti-benzo[a]pyrene-7,8-diol-9,10-oxide-DNA adducts) and shows mutagenic and carcinogenic effects. BaP is a well-known human genotoxic carcinogen (Group 1) [6]. BaP biological activity involves various molecular mechanisms, including creation of stable and depurinating DNA adducts, ROS-generating repetitive redox cycling, radical-cation mechanism and mechanism via formation of ortho-quinone, a meso-region mechanism, interaction with the aryl hydrocarbon (AhR) receptor, immunosuppression, and epigenetic changes [6,7].

The free-radical mechanism is one of major mechanisms of BaP action resulting in induction of oxidative stress in cells. The radical-cationic mechanism is associated with single-electron oxidation of BaP by cytochrome P450 (CYP) or peroxidase to form a radical cation located at carbon 6 [8]. In addition, aldo-ketoreductase enzymes oxidize the metabolite of BaP—benzo[a]pyrene-7,8-diol—to orthoquinone (benzo[a]pyrene-7,8-quinone) [9], thus contributing to the radical mechanism induced by BaP through the formation of ortho-quinone/ROS.

**Figure 1:**
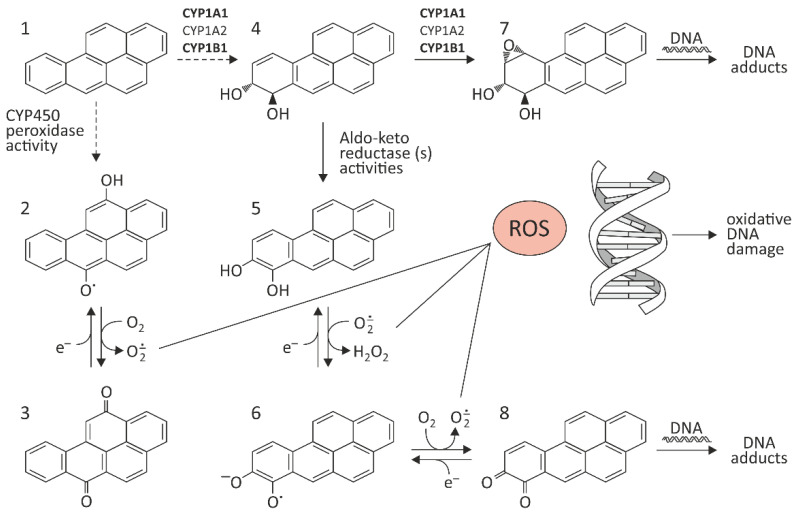
Pathway of BaP activation. The figure has been prepared from the references [8,11,12,13,14,15,16,17]. (1) BaP; (2,3) benzo[a]pyrene-diones; (4) benzo[a]pyrene-7,8-trans-dihydrodiol; (5) catechol analog; (6) semiquinone anion radicals; (7) 7β,8α-dihydroxy-9α,10α-epoxy-7,8,9,10-tetrahydrobenzo[a]pyrene (BPDE-I); (8) benzo[a]pyrene-7,8-dione.

Conversion of BaP (1) to quinone species occurs via cytochrome CYP450 peroxidase activity (Figure 1). Benzo[a]pyrene-diones (2 and 3) are formed, which can generate ROS by redox-cycling. BaP can also be biotransformed to benzo[a]pyrene-7,8-trans-dihydrodiol (4), which can be further metabolized to catechol analog (5), capable of redoxcycling to semiquinone anion radicals (6) and benzo[a]pyrene-7,8-dione (8). One of the most dangerous metabolic reactions is epoxidation of BaP by CYP450 at the 7,8 position, which results in a formation of a toxic metabolite. As mentioned above, CYP1A1, CYP1A2, and CYP1B1 can activate BaP to BaP-7,8-epoxide, which—as a result of hydration by epoxide hydrolase (EH)—is metabolized to (+/−)-B[a]P-trans-7,8-dihydrodiol (DHD). BaP-7,8-DHD may then serve as a substrate for a second CYP-dependent oxidation reaction, generating the final carcinogenic metabolite 7β,8α-dihydroxy-9α,10α-epoxy-7,8,9,10-tetrahydrobenzo[a]pyrene (BPDE-I) (7) (Figure 1). In the cell nucleus, diol-epoxides can covalently bind to DNA, forming mainly deoxyguanoside-DNA adducts, which may result in incorrect replication and mutagenesis [10].

## 2. The Effect of BaP on Oxidative Stress In Vitro and In Vivo

**Figure 2:**
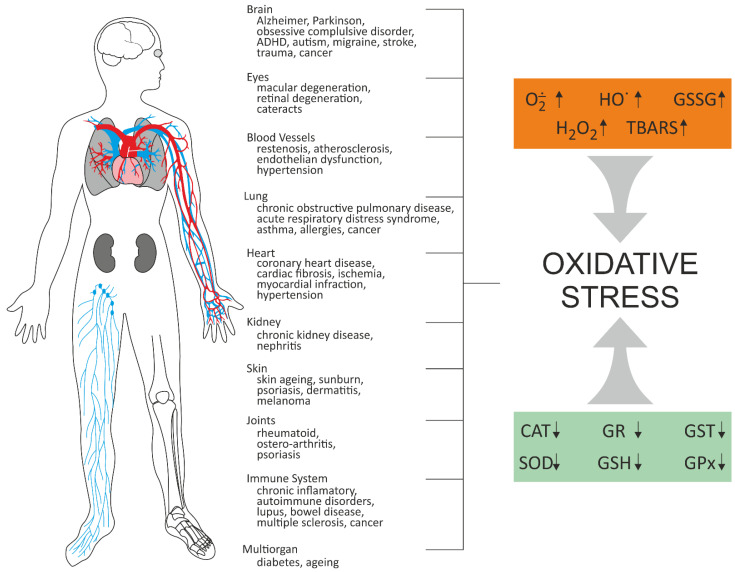
Oxidative stress-related diseases [18,19,20]. CAT–catalase; GPX–glutathione peroxidase; GR–glutathione reductase; GSH–reduced glutathione; GSSG–disulfide glutathione; GST–glutathione transferase; SOD–superoxide dismutase; TBARS–thiobarbituric acid reactive substances.

ROS are produced by several endogenous and exogenous processes, and their negative effects are neutralized by antioxidant defense. Oxidative stress occurs from the imbalance between ROS production and their elimination. Oxidative stress plays an important role in many diseases, including atherosclerosis, chronic obstructive pulmonary disease, Alzheimer disease, and cancer (Figure 2). It is known that antioxidant therapy can positively affect treatment of these diseases, and numerous small molecules possessing antioxidative effect have been shown to have a therapeutic potential in preclinical studies. The above-mentioned strategies were described in 2021 by Forman and Zhang [18] (Figure 2). Multiple antioxidant therapeutic strategies are being explored, some of which are currently undergoing clinical trials. These include: (1) Removal of O_2_**˙**^−^ before it can react with NO**˙** to form ONOO^−^ and (2) removal of H_2_O_2_ before it can form **˙**OH or HO_X_; (3) Increasing GSH level using precursors; (4) Changes in the synthesis of antioxidant enzymes, particularly through NRF2 activation; (5) Inhibition of NOXs; (6) Mitochondrial antioxidant defense; (7) Supplementing dietary antioxidants; and finally, (8) inhibition of aberrant redox signaling [18].

As shown above, BaP biotransformation may be accompanied by generation of highly reactive products. As shown above, BaP biotransformation may be accompanied by generation of highly reactive products. Additionally, BaP at presence of polyunsaturated fatty acids, such as linolenic and arachidonic acid, may undergo oxidative transformations leading to formation of lipid peroxidation products possessing a mutagenic potential [21]. Consequently, oxidative effect of BaP and its DNA adducts must be pathogenic. It is known that BaP exposure related to tobacco smoking has a role in the pathogenesis of lung and head-and-neck cancers and atherosclerosis [22]. BaP may constitute an important environmental risk factor for neurodegenerative diseases in humans [23]. Inhalation of BaP preferentially induces lung cancer [24], and oral administration preferentially induces tumors and cancers of the gastrointestinal tract [25], liver [26], and breast [27]. Petit et al. [28] recently assessed PAHs-related lung cancer risk in several industries using atmospheric concentrations of BaP as a surrogate value. They tested a total of 93 PHA-exposed groups belonging to nine industries. They showed that about 30% of exposure groups were above the maximum risk level of the European Union (10^−4^). The risk probability was >10^−3^ for coke and silicon production; >10^−4^ for the production of carbon products and aluminum; >10^−5^ for foundries and incineration processes; >10^−6^ when using lubricating oils and engine exhaust emissions; and >10^−7^ for bitumen. The probability of risk was highly variable within industries (from 1 to 1000) [28].

Recently, He et al. [29] suggested that environmental mixture of 16 priority controlled PAHs (with BaP) can induce the damages of vascular endothelial cells involved in cellular oxidative stress and inflammation.

Research showed that BaP induced ROS generation by changes in levels of hydrogen peroxide, superoxide anions, and lipoperoxides. BaP increased lipid peroxidation, induced activation of NF-κB, decreased the activity of antioxidative enzymes, such as catalase (CAT), superoxide dismutase (SOD), glutathione peroxidase (GPx), ascorbate peroxidase (AP), glutathione reductase (GR), and activated CYP450 and GST metabolizing enzymes. Ji et al. [30] evaluated effects of BaP on the function and pro-inflammatory response of human endothelial progenitor cells (EPCs) isolated from umbilical cord blood and treated with various concentrations (10, 20, and 50 µmol/L) of this substance. They demonstrated that BaP treatment significantly inhibited proliferation, migration, adhesion, and angiogenesis of EPCs in vitro. In addition, BaP induced the release of interleukin (IL)-1β and tumor necrosis factor-α from these cells. Moreover, EPCs’ exposure to BaP induced ROS generation and activation of NF-κB. In turn, Lin et al. [31] used the zebrafish model to delineate the acute toxic effects of BaP on a developing nervous system. During normal embryogenesis, low-level oxidative stress modulates neuronal development and function because ROS can regulate fundamental cellular processes. Furthermore, it is possible that oxidation of specific molecules leads to alterations in their activities, and in such a way determines the fate of a developing cell. Recent studies have provided evidence of ROS-regulating neuronal development and function, from the establishment of neuronal polarity to growth cone pathfinding; from the regulation of connectivity and synaptic transmission to the tuning of neuronal networks. In some normal physiological states, oxidative stress is known to regulate gene expression, cellular differentiation, and aging-related mechanisms, among other processes. However, Lin et al.’s experiments revealed that embryonic oxidative stress was greatly enhanced in BaP-treated embryos. Expression of CAT was decreased and sod1 expression was increased in BaP-treated embryos. These transcriptional changes were coincident with increased embryonic H_2_O_2_ and MDA levels, with the levels in BaP-treated fish similar to those in embryos treated with 120 μM H_2_O_2_.

Research on zebrafish was conducted also by Aparna and Patri [32]. They studied the impact of overcrowding stress on behavioral alterations and neurodegenerative phenotypes with an additional exposure to BaP. They investigated effects of overcrowding stress (12 fish/L) on acute waterborne exposure to BaP (0.2 mg L^−1^) in adult wild zebrafish. Among others, oxidative stress bio-markers were assayed along with histopathological changes in zebrafish brain. BaP caused an increase in lipid peroxidation and protein carbonyl formation with significant decrease of CAT activity and reduced glutathione level. Authors showed that overcrowding stress modulated the BaP-induced behavioral alterations, causing learning and memory deficiency with histopathological changes in adult zebrafish brain.

González et al. [33] showed that BaP was rapidly incorporated and metabolized in green algae *Ulva lactuca*, and induced oxidative stress and activation of antioxidant enzymes, as well as CYP450 and GST metabolizing enzymes in tested organism. BaP induced oxidative stress by changes in levels of hydrogen peroxide, superoxide anions, and lipoperoxides, as well as changes in activities of antioxidant enzymes: SOD, CAT, AP, GR, and GPx. Recently, Gao et al. [34] pointed to an important BaP-induced formation of ROS by an effect on ROS/HIF-1α/HO-1 signaling (heme oxygenase 1–HO-1). The authors analyzed the effect of BaP (1.5 or 25 µM) exposure on A549 and MCF-7 cancer cells and observed induction of ROS and modulation of HIF-1α and HO-1 in A549 and MCF-7 cancer cells. However, Zhu et al. [35] showed that BaP-induced cytotoxicity in human lung epithelial cells (BEAS-2B) occurred through DNA damage, cell cycle arrest, ROS production, modulation of metabolizing enzymes, and expression/activation of p53, PARP-1, survivin, and Bax/Bcl-2.

Also, Ranjit et al. [36] suggested that BaP enhanced HIV-1 replication in macrophages by a CYP-mediated oxidative stress pathway followed by the NF-κB pathway. They observed a ~3 to 4-fold increase in HIV-1 replication in U1 cells and human primary macrophages following a chronic BaP exposure. CYP1A1 in turn metabolizes BaP into various BaP-metabolites, during which large amounts of ROS are generated. The resulting oxidative stress induces HIV-1 replication in cells via a NF-κB pathway. They observed a ~30-fold increase in the expression of *CYP1A1* gene at mRNA level, a ~2.5-fold increase in its enzymatic activity, as well as elevated ROS and cytotoxicity in U1 cells. Knock-down of the *CYP1A1* gene using siRNA and treatment with selective CYP inhibitors and antioxidants significantly reduced HIV-1 replication. Further, they observed a nuclear translocation of NF-κB subunits (p50 and p65) following a chronic BaP exposure, which was reduced by treatment with siRNA and antioxidants/CYP inhibitors.

Numerous studies have indicated oxidative changes caused by BaP both in vitro [30,34,35,36] and in vivo [31,32,33,37] (Table 1).

BaP is widely known to cause DNA and protein damage, which is closely related to cell transformation. Accordingly, studies on natural bioactive compounds that attenuate such chemical-induced toxicities have significant impacts on public health.

The purpose of this paper is to provide a review of literature on protective properties of selected substances against oxidative effects of BaP. Toxic effects of BaP, including those that are carcinogenic, may be reduced by the use of antioxidant. According to recent studies, these adverse effects are eliminated by substances, such as vitamin E [35,37], curcumin [37,38], baicalein [39], hesperetin [40], myricetin [41], resveratrol [42], rhaponticin [43], taurine [44], compounds contained in green and white tea [45], atorvastatin [46], and diallyl sulfide [47].

## 3. Protective Properties of Selected Substances against Oxidative Effects of BaP

### 3.1. Vitamin E

Vitamin E (α-Tocopherol) is the main fat-soluble antioxidant in the body. As an antioxidant, it acts in cell membranes, where it prevents the spread of free radical reactions, although it has also been shown to have a pro-oxidative effect. Free-radical scavenging reactions of α-tocopherol take place via the α-tocopheroxyl radical as an intermediate [48].

The antioxidant properties of vitamin E are exerted through its phenolic hydroxyl group, which donates hydrogen to peroxyl radicals, resulting in the formation of stable lipid species. α-Tocopherol regulates signal transduction cascades not only at the mRNA, but also at the miRNA level since miRNA 122a (involved in lipid metabolism) and miRNA 125b (involved in inflammation) are downregulated by α-tocopherol [49].

Lin et al. [37] investigated a direct effect of maternal BaP exposure on closure of the neural tube in mice. They found that BaP significantly increased expression of *CYP1A1*, *SOD1,* and *SOD2* gene, while repressing *GPX1* gene. Adding vitamin E to food given to BaP-exposed mice significantly protected them against neural tube defects. The researchers showed that vitamin E partially normalized gene expression associated with oxidative stress as well as with excessive apoptosis. In other investigations provided in normal human lung epithelial cells (BEAS-2B) [35], BaP increased ROS level and BPDE-I DNA adducts formation as well as increased the ratio of Bax/Bcl-2, and these changes were reversed by vitamin E.

### 3.2. Polyphenols

Polyphenols can be used in the prevention and control of different diseases by removing ROS and regulating the oxidative stress in the human body. This is due to the phenolic hydroxyl structure of these substances, in which the electrons have a conjugation effect; the hydrogen ion’s binding ability is weakened and, therefore, more likely to be dissociated, so the active hydrogen ion neutralizes the organic radicals and ROS, scavenging the free radicals. Polyphenols react with ROS to form relatively stable phenolic oxygen radicals. In addition to directly scavenging free radicals, polyphenols protect the body from oxidative damage by regulating different types of oxidase and antioxidant enzyme activities. Polyphenols can protect cells from oxidative damage by regulating certain cell signaling pathways (e.g., NRF2-KEAP1-ARE or MAPK) and can increase the expression of antioxidant enzymes, such as NQO1 and GST [50].

#### 3.2.1. Curcumin

**Figure 3:**
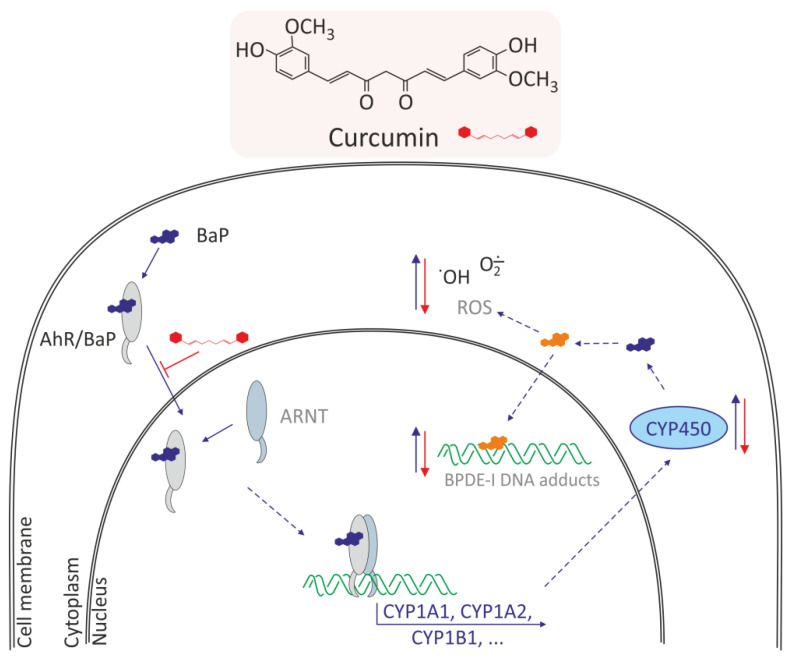
Mechanism of antioxidative effects of curcumin in normal human lung epithelial cells and HepG2 cells [35,38,53]. Curcumin induces: decrease of AhR translocation into nuclei, decrease of CYP450 family expression, decrease metabolism of BaP, and in consequence decreases the BPDE-I DNA adducts and ROS level.

Curcumin (1,7-bis-(4-hydroxy-3-methoxyphenyl)-1,6-heptadieno-3,5-dione) also known as diferuloylmethane belongs to hydrophobic polyphenols containing two ferulic acid residues joined by a methylene bridge. Curcumin has anti-inflammatory and antioxidant activity [51]. Curcumin belong curcuminoids, which are contained in turmeric (*Curcuma longa* L.), a perennial plant belonging to the ginger family. Because of its color, it is also called Indian saffron. It grows wild in India and is cultivated in many countries with tropical climates, such as India, Pakistan, China, and Haiti. This plant also produces other secondary metabolites, such as phenolic acids, flavonoids, alkaloids, terpenoids, tannins, and saponins, whose biological properties have been known for centuries [52]. The mechanism of curcumin in modulating the lipid accumulation and oxidative stress mediated by BaP cytotoxicity in HepG2 cells was analyzed [38]. The study indicated that curcumin recovered cell viability and suppressed BaP-induced lipid accumulation and ROS generation, which could potentially induce nonalcoholic fatty liver disease. Curcumin induced cytochrome P450 family downregulation resulting from decreased AhR translocation into nuclei attenuated effects of BaP-induced lipid accumulation and repressed cell viability. Moreover, curcumin-induced reduction in ROS generation decreased the nuclear translocation of nuclear factor erythroid-2-related factor 2 (NRF2) and expression of phase-II detoxifying enzymes (Figure 3).

Zhu et al. [35] investigated potential protective effects of curcumin and vitamin E on BaP-induced changes in normal human lung epithelial cells (BEAS-2B). The researchers showed that these compounds prevented cells from BaP-induced cell cycle arrest and growth inhibition, significantly suppressed BaP-induced ROS levels, and decreased BPDE-I DNA adducts. Additionally, BaP increased the level of activated p53 and PARP-1, whereas this increase was markedly prevented by co-treatment with curcumin and vitamin E. Survivin expression was decreased by BaP, and significantly restored by curcumin. BaP also increased the ratio of Bax/Bcl-2, and this increase was reversed by vitamin E. Curcumin and vitamin E also reversed BaP-induced oxidative alterations, and therefore they may be effective natural remedies on adverse effects of BaP in lung cells (Figure 3).

#### 3.2.2. Resveratrol and Other Polyphenols

Omidian et al. [54] investigated protective effects of various polyphenols (resveratrol, quercetin, catechin, cyanidin, kuromanin, and berberine) against BaP-induced oxidative stress and neoplastic transformation in the Bhas 42 cell carcinogenesis assay.

Polyphenols, and especially resveratrol, are found naturally in fruits, nuts, flowers, seeds, and bark of various plants, and are an integral part of the human diet [55].

All of the investigated polyphenols completely prevented the increased intracellular ROS generation by BaP at 12 h, and mostly inhibited it after three days. BaP increased mitochondrial superoxide generation at 12 h, and this effect was inhibited by anthocyanins and berberine. BaP increased the expression of genes related to oxidative stress and inflammation (NRF2, UCP2, and TNF-α) after 24 h. Polyphenols strongly inhibited the increase in TNF-α formation, and also inhibited the increase in UCP2 formation. All tested polyphenols were able to inhibit BaP-induced increase in markers of oxidative stress and inflammation, and inhibited cellular transformation, with resveratrol being notable for the strongest preventive effect on cell transformation. These results support the role of dietary polyphenols in protection against BaP-induced carcinogenesis.

**Figure 4:**
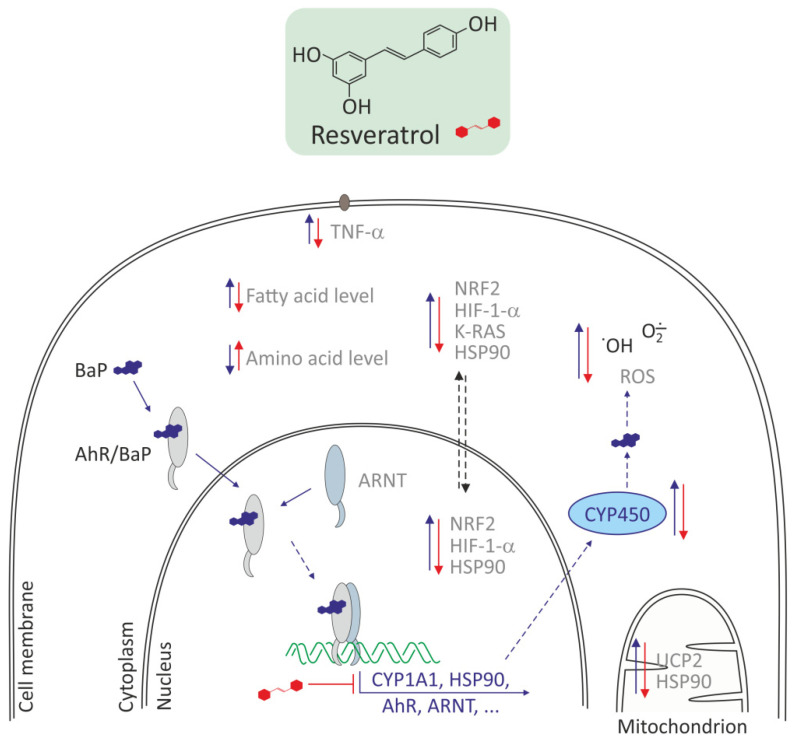
Antioxidative effects of resveratrol in Bhas 42 and in human lung epithelial cells (BEAS-2B)s [42,54,55]. Resveratrol decreases CYP450 family, HSP90, AhR, ARNT expression, downstream regulatory factors, such as NRF2, K-RAS, HIF-1-α, TNF-α, HSP90, and in consequence decreases metabolism of BaP and ROS level. Resveratrol increases the level of amino acid and decreases the level of fatty acid.

Similarly, Gao et al. [42] showed that resveratrol could reduce toxic effects of BaP. The use of BaP at 5 µg/L promoted invasion and migration of BEAS-2B cells. Expression levels of AhR mRNA and protein and its target genes involved in BaP metabolism, such as the AhR nuclear translocator, heat shock protein 90, and CYP1A1, were significantly increased upon exposure to BaP. mRNA expression levels of downstream regulatory factors related to both AhR and EMT signaling pathways, such as NRF2, K-RAS, and hypoxia-induced factor 1-alpha (HIF-1-α), increased significantly. The above changes induced by BaP were significantly attenuated or even arrested by resveratrol. A beneficial effect of resveratrol on BEAS-2B lung cells exposed to BaP was noted by Ye et al. [56]. These authors showed that amino acid and fatty acid metabolites were significantly altered in BEAS-2B cells exposed to BaP and responded to the action of resveratrol. Pathway analysis showed that 30 metabolic pathways altered by BaP were significantly modulated in response to resveratrol intervention. Moreover, most of amino acid levels were significantly lowered, while most of fatty acid levels were significantly elevated in BEAS-2B cells exposed to BaP, and the above changes were abolished by resveratrol intervention (Figure 4). Ye at al. revealed AhR mediated changes in the levels of metabolites involved in amino acid, lipid, carbohydrate, and nucleotide metabolism in BaP-exposed BEAS-2B cells. They discovered that peroxisome proliferator-activated receptor-g (PPAR/G) signaling was suppressed, while the fatty acid import by fatty acid transport protein 1 (FATP1) was activated, thus triggering fatty acid accumulation in BaP exposed BEAS-2B cells, and the above changes were abolished by resveratrol intervention. These data suggested amino acid and fatty acid metabolism regulated by AHR and PPAR-FATP1 signaling as potential therapeutic targets for intervening BaP-induced toxicity and related diseases.

Also, Çelik et al. [57] showed that resveratrol could reverse oxidative alterations induced by BaP. INS-1 (832/13) insulinoma cells were treated with BaP 20 at μM for 24 h after 48 h of initial preconditioning with resveratrol at 10 μM. Oxidative stress status, insulin secretion and apoptosis were analyzed. Resveratrol increased GSH to the control level and elevated the insulin concentration of culture medium.

#### 3.2.3. Polyphenols from Green and White Tea

**Figure 5:**
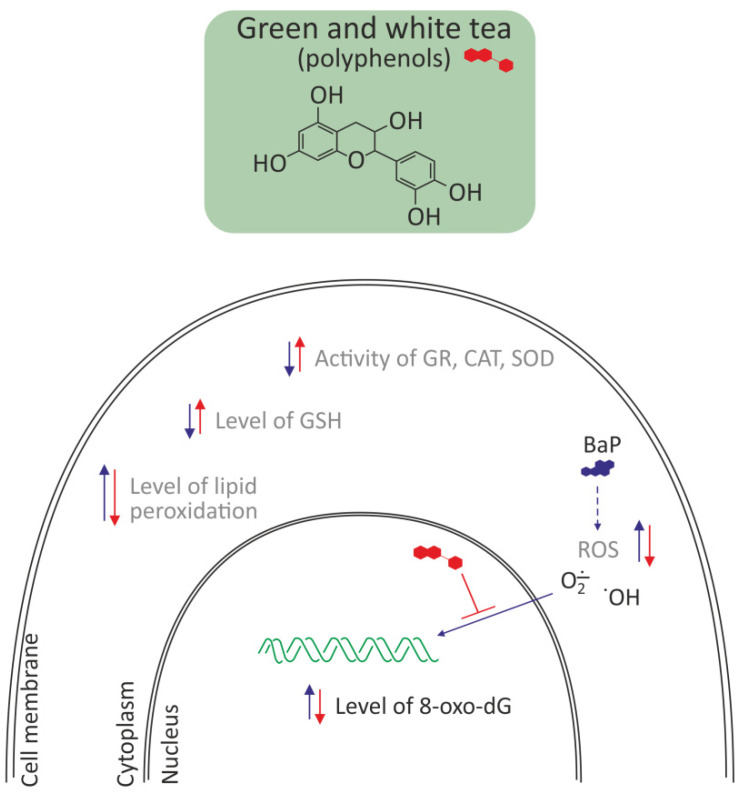
Antioxidative effects of polyphenols from green and white tea in liver and lung of Balb/c mice [45,58]. Polyphenols decreased oxidative stress by removing ROS, and in consequence decreased the level of 8-oxo-dG and lipid peroxidation and increased the level of reduced glutathione. They also increased the activity of antioxidative enzymes, like GR, CAT, and SOD.

The most important tea polyphenols are epigallocatechin-3-gallate, epicatechin-3-gallate, epigallocatechin, and epicatechin [58]. Kumar et al. [45] investigated the protective effect of green tea and white tea against BaP-induced oxidative stress and DNA damage in livers and lungs of Balb/c mice. A single dose of BaP (125 mg/kg, b.w.) increased the level of lipid peroxidation (LPO) and significantly decreased endogenous antioxidants such as SOD, GR, CAT, and GSH. Pretreatment with green tea and white tea for 35 days before a single dose of BaP restored the decreased activity of GR, SOD, and CAT in liver and also tended to normalize levels of GSH and LPO in both hepatic and pulmonary tissues. Green tea and white tea also decreased the percentage of damaged DNA and 8-hydroxy-2-deoxyguanosine levels. Antioxidant activity of green tea and white tea was effective in combating BaP-induced oxidative damage and DNA lesions. White tea was found to offer a better protection than green tea with respect to CAT, DNA damage level, GST activity, and GSH content (Figure 5). The overall concentration of polyphenols, total catechins, gallic acid, theobromine, epigallocatechin, and epigallocatechin-3-gallate are significantly lower in green tea compared to white tea [58].

#### 3.2.4. Flawonoids

##### Baicalein

Baicalein (5,6,7-trihydroxyflavone) is a flavone, originally isolated from the roots of *Scutellaria baicalensis* and *Scutellaria lateriflora*. It is also reported in *Oroxylum indicum* or Indian trumpet flower. It is the aglycone of baicalin. Baicalein and baicalin are the compounds belonging to the flavone subfamily of flavonoids [39].

Baicalein, as the anti-inflammatory agent, inhibits expression of CYP1A1, which significantly reduces the oxidizing effect of BaP. Tanaka et al. [39] treated normal human epidermal keratinocytes and HaCaT keratinocytes with compounds such as: baicalein, BaP, or baicalein/BaP mixture and assessed the following: CYP1A1 expression, antioxidant pathways, ROS production, and expression of pro-inflammatory cytokines. Preparations containing baicalein and baicalein Wogon and Oren-gedoku-to inhibited BaP-induced expression of CYP1A1. In addition, baicalein activated the antioxidant system nuclear factor 2 associated with erythroid 2 (NRF2) and heme oxygenase 1 (HMOX1), leading to a reduction in BaP-induced ROS production. Moreover, levels of IL1A and IL1B induced by BaP were reduced by baicalein. Baicalein inhibited phosphorylation of Src, a component of the cytoplasmic AhR complex that ultimately disrupted the cytoplasmic-to-nucleus translocation of AhR. These results indicate that herbal medicines containing baicalein and baicalein itself may be helpful in alleviating of toxic effects provoked by BaP through the dual action of inhibiting AhR-CYP1A1 and activating NRF2-HMOX1 [39].

##### Myricetin

Myricetin, a 3,3′,4′,5,5′,7-hexahydroxyflavone belongs to flavanols (flavonoids, polyphenols). Myricetin is mainly present in the glycoside form (O-glycosides), in vegetables, fruits, nuts, berries, herbs, and beverages, such as tea or wine. The myricetin was identified in the plants, such as *Rosa canina* L. (rosa hip), *Urtica dioica* L. (nettle), and *Portulaca oleracea* L. (purslane). Myricetin is the most abundant flavanol of black currant as well as it is present in high concentrations in honey [59].

In turn, Jee et al. [41] examined protective effects of myricetin against BaP-induced toxicity. They found that co-administration of myricetin with BaP reduced formation of BaP metabolite, i.e., BaP-7,8-dihydrodiol-9,10-epoxide (BPDE-I), and ultimately formation of 8-hydroxy-2-deoxyguanosine (8-OHdG) and BPDE-I DNA in the liver, kidneys, colon, and stomach. This inhibition was more pronounced in hepatic than in other tissues. Myricetin mainly reduced *CYP1A1* expression and induced glutathione S-transferase activity that determined BaP metabolism and formation of DNA-conjugated metabolite of BaP. In conclusion, myricetin attenuated BaP-induced genotoxicity by regulating phase I and II enzymes activities.

##### Catechin Hydrate

Khattab et al. [60] aimed to evaluate the protective effect of catechin hydrate on BaP-induced toxicity in lungs of adult albino rats. Catechin hydrate belongs to the group of flavan-3-ols, belonging to the chemical family of flavonoids. These authors showed that BaP caused a decrease in the activity of antioxidant enzymes (SOD, CAT) and an increase in the level of malondialdehyde (MDA). In addition, BaP induced DNA damage and activated the apoptotic pathway by upregulating expression of BAX and caspase-3 genes and downregulating BCL-2 genes. However, treatment with catechin hydrate increased the activity of antioxidant enzymes and also regulated apoptosis. Perceptible histological changes in the lungs also confirmed the protective effect of catechin hydrate.

##### Hesperetin

Hesperetin is a natural flavanone and an aglycone of hesperidin. This compound is present in citrus fruits, i.e., lemon and orange. Hesperetin shows various beneficial effects, including antioxidant, anti-inflammatory, and chemopreventive. It also has anti-carcinogenic properties [61,62].

Lung cancer is the major cause of cancer mortality and is a growing economic burden worldwide. Chemoprevention, employing the use of natural, dietary, or synthetic agents has become an appealing strategy to combat the increasing worldwide incidence of cancers. Bodduluru et al. [40] analyzed the chemopreventive potential of hesperetin by estimating the status of lipid peroxidation, levels of enzymic and nonenzymic antioxidants, proinflammatory cytokines, and histopathology of pulmonary tissues of control and experimental Swiss albino mice. Their study was designed to investigate the mechanism-based chemopreventive nature of hesperetin against BaP-induced lung carcinogenesis. Administration of BaP (50 mg/kg, P.O.) resulted in an increase in lung weight, LPO with concomitant decrease in body weight, enzymic (SOD, CAT, GPx, GR, and GST), and non-enzymic (GSH, Vit C and Vit E) antioxidants. Further, elevated levels of TNF-α along with activated expression of *NF-κB*, *PCNA*, and *CYP1A1*, and downregulation of *NRF2* were observed in BaP-intoxicated animals. Pre- and post-treatment with hesperetin effectively suppressed BaP-induced lung carcinoma and the associated precancerous lesions by alleviating LPO, modulating antioxidants and decreasing expressions of *NF-κB*, *PCNA*, and *CYP1A1*. These findings demonstrate that hesperetin has a chemopreventive potential against BaP-induced lung cancer. This potential is attributed to its free radical scavenging, antioxidant, anti-inflammatory, and antiproliferative properties.

### 3.3. Rhaponticin

Rhaponticin (3,3′,5-trihydroxy-4′-methoxystilbene 3-O-β-d-glucoside; synonym–rhapontin) is a stilbene compound, mainly found in various species of rhubarb (*Rheum* L.). The aglycone, rhapontigenin, is thought to be a biologically active form of rhaponticin [63].

Wang et al. [43] analyzed antioxidant and antitumor effects of rhaponticin, a stilbenoid glucoside. Rhaponticin is a compound isolated from various herbs, including *Rheum undulatum*, common in Asia. Lung cancer tumor in mice was induced with BaP at 50 mg/kg body weight (*w*/*w*) after oral administration for six weeks (twice/week). Rhaponticin was administered orally at 30 mg/kg body weight (twice/week) to BaP-exposed mice from week 12 to 18. The results showed that BaP-exposed mice had decreased body weight, increased lung weight, increased levels of tumor markers, such as aryl hydrocarbon hydroxylase (AHH) and lactate dehydrogenase (LDH), and increased levels of pro-inflammatory cytokines. Activities of CAT and SOD were reduced and lipid peroxidation increased in immune cells compared to control cells. Administration of rhaponticin improved the above parameters, as well as reduced histopathological changes in pulmonary tissue.

### 3.4. Taurine

BaP is toxic to the reproductive system [64,65]. Recent studies have shown that taurine (2-aminoethanesulfonic acid), an organic chemical compound from the group of biogenic amino acids, ameliorated toxic reactions in the epididymis and testes of rats exposed to BaP. Rats were simultaneously administered with BaP at 10 mg/kg and taurine at 100 and 200 mg/kg for 28 consecutive days. It has been shown that taurine significantly alleviated toxic effects induced by BaP, such as decreased sperm quality, decreased levels of reproductive hormones, as well as increased levels of oxidative stress and inflammatory biomarkers. Taurine also reduced BaP-damaging effects within epididymis and testicles in rats [44].

### 3.5. Atorvastatin

It is suggested in published studies that oxidative stress expansion can lead to lung cancer. Du et al. [46] analyzed a possible inhibitory effect of atorvastatin against BaP-induced lung cancer, as well as inhibitory effect of atorvastatin on BaP induction of lung cancer in experimental rats. They studied cytotoxic effects of drug atorvastatin (5, 10, and 20 mg/kg body weight) on lung cancer H460 and A549 cell lines, as well as cell cycle arrest and cell morphology. These authors showed that cell cycle arrest occurred at the G2/M phase after atorvastatin treatment. In rats, atorvastatin increased cytochrome C expression and caspase activity in a dose-dependent manner, and increased the activity of antioxidative enzymes, including GPx, SOD, GST, and CAT as well as increased the level of reduced glutathione. This compound also reduced the level of nitrate and LPO.

### 3.6. Organosulfur Compounds of Garlic

**Figure 6:**
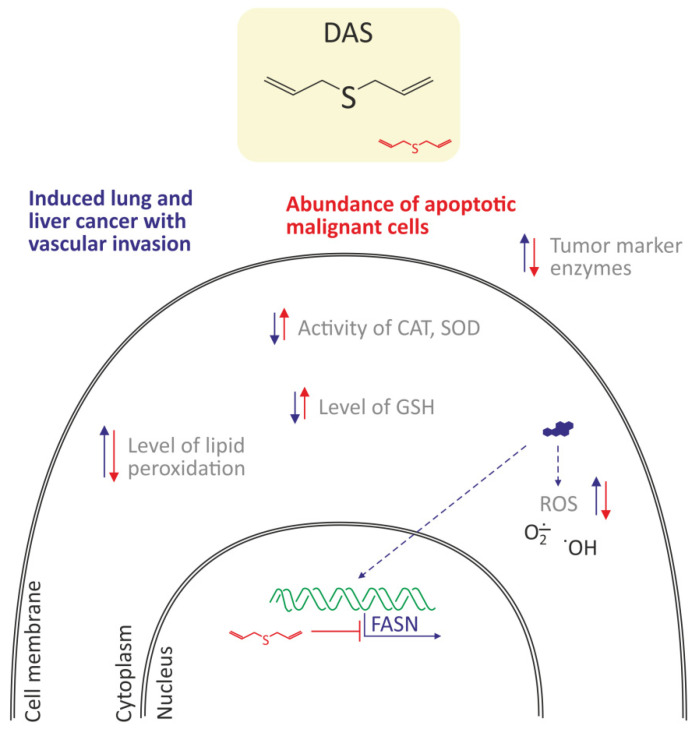
Antioxidant and anticancer properties of diallyl sulfide (DAS) in lung cancer cells in a murine model [47]. DAS decreased oxidative stress by a decrease of ROS level, and lipid peroxidation as well as increased the level of reduced glutathione and activities of CAT and SOD. Moreover, decreased expression of fatty acid synthase (FASN), which led to apoptosis of cancer cells was noted.

Khan et al. [47] showed that diallyl sulfide (DAS), one of the organo-sulfur secondary metabolites in garlic, has been shown to inhibit the proliferation of BaP-induced cancer cells lung in a murine model. The mice were exposed to 50 mg/kg of BaP twice a week for four weeks in order to induce lung carcinoma. Pretreatment of mice with DAS (100 mg/kg) was started two weeks before BaP exposure and further continued for 21 weeks. The authors observed on the basis of histopathological examinations that DAS prevented the progression of malignant lung cancer and metastasis in the liver. DAS decreased the activity of tumor marker enzymes (ADA, AHH, γ-GT, LDH) as well as increased antioxidant enzymes, SOD, and CAT, in BaP-exposed mice. DAS also induced apoptosis and decreased cellular ROS level in mice cancer cells treated with BaP. It is known that the expression of fatty acid synthase is up-regulated in the carcinogen-induced lung cancer model [66]. Khan et al. [47] also detected high level of fatty acid synthase (FASN) in the lungs and liver sections of the BaP-exposed mice and a significant decrease in its expression was recorded in the tested model. Additionally, several studies including the molecular docking, suggested the cholesterol-lowering effect of garlic and organosulfur compounds by inhibiting FASN [67,68]. The down-regulation of FASN, leading to apoptosis, was maybe one of the molecular events responsible for the chemopreventive effect of DAS (Figure 6).

## 4. Summary

BaP is a well-known carcinogen found in the natural environment and in some foods, particularly grilled ones. Therefore, human exposure to this compound is common, which is disturbing taken into consideration that numerous diseases may be associated with its action.

Summing up, BaP induces disruptions in redox status and favors emergence of oxidative stress; decreases activity of antioxidant enzymes such as CAT, GPx, SOD, GST, and GR by changes in their expression; decreases the level of GSH, Vit C, and Vit E; increases the lipid peroxidation and protein carbonylation; increases CYP1A1 expression and expression of pro-inflammatory cytokines. BaP metabolites (mainly BPDE-I) and the resulting ROS formation are mainly responsible for the oxidative effect of BaP.

Various studies have assessed the ability of biologically active molecules to withstand strong oxidative effects of BaP. As shown in this review, the compounds, such as vitamin E, curcumin, quercetin, catechin, cyanidin, kuromanin, berberine, resveratrol, baicalein, myricetin, catechin hydrate, hesperetin, rhaponticin, taurine, atorvastatin, as well as substances contained in green and white tea are capable of reducing BaP-induced oxidative stress (Table 2).

As outlined above, a number of compounds act to prevent the oxidative stress caused by BaP or to reduce the level of ROS. Polyphenols, such as curcumin and resveratrol seem to be particularly promising. Unfortunately, studies concern relatively high concentrations of antioxidants, which in the case of polyphenols are difficult to achieve in cells in vivo. For example, despite encouraging evidence, the therapeutic use of curcumin is limited due to its well-known poor aqueous solubility and low bioavailability. Therefore, the encapsulation of curcumin in polymeric nanocarriers has been proposed as an alternative way to overcome problems related to its physicochemical properties [69]. This effect can be improved by co-encapsulating this polyphenol with another substance with biological activity. The problem concerning polyphenols bioavailability should seriously be taken into account, if they are to be used for human health. Research on these compounds should therefore focus on developing the carriers for these substances that allow them to achieve appropriate target concentration, which would be crucial in preventing humans from toxic effects exerted by BaP.

## 5. Conclusions

The compounds that are able to reduce toxic effects exerted by BaP act through various mechanisms:

1. Decrease of AhR translocation (e.g., resveratrol, curcumin)
2. Downregulation of Cyp 450 level and other proteins involved in BaP metabolism (e.g., resveratrol, curcumin)
3. Decrease of BPDA-DNA adducts and other DNA damage formation (e.g., curcumin; polyphenols from white and green tea)
4. Decrease of expression of genes related to oxidative stress and inflammation (e.g., hesperetin, rhaponticin, curcumin, taurine, atorvastatin)
5. Increase of expression and activity on antioxidative enzymes and antioxidants (e.g., vitamin E, diallyl sulfide, hesperetin)
6. Decrease of fatty acid synthase (FASN), and in consequence decrease of fatty acid level in the liver (resveratrol, DAS)
7. Decrease of ROS level by binding of hydrogen ions and scavenging free radicals (e.g., polyphenols from white and green tea, curcumin, resveratrol)

These compounds prevent cell/organism from oxidative stress provoked by BaP (conclusions 1–6) and reduce it through scavenging previously formed free radicals.

## Figures and Tables

**Table 1:** Oxidative properties of BaP studies of oxidative stress induced by benzo[a]pyrene.

BaP Concentrations	Cells/ Organisms	Oxidative Effects Induced by BaP	References
10; 20; 50 µM	Human endothelial progenitor cells (EPCs)	Release of IL-1β and TNF-α from these cells	[30]
1; 5 µM	A549 and MCF-7 cancer cells	Formation of ROS by influencing ROS/HIF-1α/HO-1 signaling pathway	[34]
5 µM	Human lung epithelial cells (BEAS-2B)	ROS production and DNA damage	[35]
100 nM; 1 µM	Human monocytes and U937 cells	ROS production, increase in the expression of *CYP1A1* gene, and induction of NF-κB pathway	[36]
250 mg kg^–1^	Mice–maternal exposure to BaP	Increased expression of *CYP1A1*, *SOD1*, and *SOD2* gene, and repressed *GPX1* gene	[37]
10; 20 µM	Zebrafish embryos	Decrease of *CAT* and *SOD1* gene expression, increase H_2_O_2_ and MDA level	[31]
0.2 mg L^−1^	Adult zebrafish	Increase of lipid peroxidation and protein carbonyl formation, decrease CAT activity and reduce GSH level	[32]
5 µM	Green algae (*Ulva lactuca*)	Changes in the level of ROS, changes in SOD, CAT, AP, GR, GPX activities	[33]

**Table 2:** Protective properties of selected substances against oxidative effects of BaP in in vivo and in vitro studies.

BaP Concentrations	Cells/Organisms	Oxidative Effects Induced by BaP	Compounds That Reduce the Oxidative Effect of BaP	References
250 mg kg^–1^	Female mice	Increased expression of *CYP1A1, SOD1,* and *SOD2*; decreased *GPX1*	Vitamin E 0.125%, *w*/*w*	[37]
0–80 µM; 5 µM	Human lung epithelial cells (BEAS-2B)	ROS production	Vitamin E and curumine 0–80 µM	[35]
10 µM	HepG2 cells	Lipid accumulation and oxidative stress	Curcumin 1; 5; 10; 20; 40 µM	[38]
4 µM	Bhas 42 cells	Increased expression of NRF2, UCP2, TNF-α	Polyphenols 5 µM	[54]
1.28; 6.4; 32; 160; 800; 4000; 20000 μg/L	BEAS-2B lung cells	Changes in expression levels of AhR mRNA and genes involved in BaP metabolism	Resveratrol 0.1; 0.2; 0.4; 0.8; 1; 3.2; 6.4 μg/mL	[42]
5 µg/L	BEAS-2B lung cells	Changes in 30 metabolic pathways	Resveratrol 0.1 mg/L	[56]
20 μM	INS-1 (832/13) insulinoma cells	Decrease of GSH level and antioxidant status	Resveratrol 80 μM	[57]
1 μM	NHEKs epidermal keratinocytes and HaCaT keratinocytes	Increase in *CYP1A1* expression, ROS production and expression of pro-inflammatory cytokines	Baicalein 10 µM for NHEKs and 25 µM for HaCaT cells	[39]
125 mg/kg b.w. orally	Balb/c mice	Increased LPO level and decrease in antioxidants level	Green tea and white tea−2%	[45]
1; 2.5; 5; 10 μM 2 mg/kg	HepG2 cells rats	Oxidative damage to DNA and increase of CYP1A1 expression	Myricetin 15 mg/kg	[41]
50 mg/kg 1/20 of LD50	Adult albino rats	Decrease in the activity of SOD, CAT, and an increase in MDA level	Catechin hydrate 20 mg/kg body weight	[58]
50 mg/kg p.o.	Swiss albino mice	Increase in LPO and decrease in SOD, CAT, GPx, GR, and GST activities and decrease in GSH, Vit C, and Vit E levels	Hesperetin 50 mg/kg body weight	[40]
50 mg/kg body weight	Mice	Decrease of CAT and SOD activities and increase in LPO level	Rhaponticin 30 mg/kg body weight	[43]
10 mg/kg	Rats	Oxidative stress and high level of inflammatory biomarkers	Taurine 100, 200 mg/kg	[44]
50 mg/kg, body weight	Swiss Wistar rats	Decreased GPx, SOD, GST activities, and GSH level, increased nitrate and LPO levels	Atorvastatin 5, 10, 20 mg/kg body weight	[46]
50 mg/kg of BaP twice a week for 4 weeks	Mice	Increased activity of enzymatic tumor markers: ADA, AHH, γ-GT, LDH, and decreased activity of antioxidant enzymes: SOD and CAT. High level of fatty acid synthase	Diallyl sulfide 100 mg/kg body weight	[47]

## Data Availability

Not applicable.

## Sample Availability

Samples of the compounds are not available from the authors.

## Abbreviations

8-OHdG–8-hydroxy-2-deoxyguanosine; AHH–aryl hydrocarbon hydroxylase; AhR–aryl hydrocarbon receptor; AP–ascorbate peroxidase; BEAS-2B–Human lung epithelial cells; BEAS-2B–human lung epithelial cells; BaP–benzo[a]pyrene; BPDE-I–7β,8α-dihydroxy-9α,10α-epoxy-7,8,9,10-tetrahydrobenzo[a]pyrene; CAT–catalase; *CAT*–gene of catalase; CYP–cytochrome P450; DHD–(+/−)-B[a]P-trans-7,8-dihydrodiol; EH–epoxide hydrolase; EPCs–human endothelial progenitor cells; FATP1–fatty acid transport protein 1; GPx–glutathione peroxidase; GPX1–of glutathione peroxidase 1; GR–glutathione reductase; GSH–reduced glutathione; GSSG–disulfide glutathione; GST–glutathione transferase; HMOX1–heme oxygenase 1; NRF2–nuclear factor erythroid-2-related factor 2; LDH–lactate dehydrogenase; LPO–lipid peroxidation; MDA–malondialdehyde levels; PAH–polycyclic aromatic hydrocarbon; *PCNA*–gen of Proliferating Cell Nuclear Antigen; PPAR/G–peroxisome proliferator-activated receptor-g; ROS–reactive oxygen species; SOD–superoxide dismutase; *SOD1*–gene of superoxide dismutase 1; *SOD2*–gene of superoxide dismutase 2; SOD–superoxide dismutase; TBARS–thiobarbituric acid reactive substances; Vit C–vitamin C; Vit E–vitamin E; UCP2–gene of Uncoupling Protein 2.
